# An integrated photocatalytic/enzymatic system for the reduction of CO_2_ to methanol in bioglycerol–water

**DOI:** 10.3762/bjoc.10.267

**Published:** 2014-11-03

**Authors:** Michele Aresta, Angela Dibenedetto, Tomasz Baran, Antonella Angelini, Przemysław Łabuz, Wojciech Macyk

**Affiliations:** 1 Chemical and Biomolecular Engineering Department, NUS, 4 Engineering Drive 4, Singapore 117585-SG; 2 IC2R srl Tecnopolis, km 3 via Casamassima, 70018 Valenzano (BA), Italy; 3 CIRCC, Via Celso Ulpiani 27, 70126 Bari, Italy; 4 Department of Chemistry, University of Bari, Via Orabona 4, 70125 Bari, Italy; 5 Faculty of Chemistry Jagiellonian University Ingardena 3, 30-060 Kraków, Poland

**Keywords:** CO_2_ chemistry, electron transfer, enzymatic CO_2_ reduction, NADH regeneration, photochemistry, photosensitization

## Abstract

A hybrid enzymatic/photocatalytic approach for the conversion of CO_2_ into methanol is described. For the approach discussed here, the production of one mol of CH_3_OH from CO_2_ requires three enzymes and the consumption of three mol of NADH. Regeneration of the cofactor NADH from NAD^+^ was achieved by using visible-light-active, heterogeneous, TiO_2_-based photocatalysts. The efficiency of the regeneration process is enhanced by using a Rh(III)-complex for facilitating the electron and hydride transfer from the H-donor (water or a water–glycerol solution) to NAD^+^. This resulted in the production of 100 to 1000 mol of CH_3_OH from one mol of NADH, providing the possibility for practical application.

## Introduction

The reduction of CO_2_ to fuel is a technology that could contribute to the recycling of large quantities of carbon. Among the various routes, the enzymatic reduction of CO_2_ in inexpensive H-donor solvents to produce methanol (or other C_1_ molecules) is an attractive option. Redox enzymes are of industrial interest as they may catalyze reactions in which the use of conventional chemical catalysts is restricted [1]. Unfortunately, their application is quite limited due to the high cost of their cofactors. A huge effort is being made for the in situ regeneration of these cofactors using various approaches such as: the use of secondary enzymes, electrochemical regeneration, or even the use of living cells. Noteworthy is that the regeneration of the cofactor often involves the potential production of a variety of isomers of the active species or even the formation of dimers that may not be active in promoting the enzymatic reaction or may even act as inhibitors. In nature, cofactors are usually regenerated via enzymatic reactions. One of the most interesting cofactors is nicotinamide adenine dinucleotide (NAD^+^), a cofactor of the oxydoreductase class of enzymes. NAD^+^, together with its reduced form, 1,4-NADH, plays an essential role in many metabolic processes of living cells. NADH is also important in industrial biocatalysis, namely in the process of reductive synthesis of chiral organic compounds [2–3].

**Figure 1:**
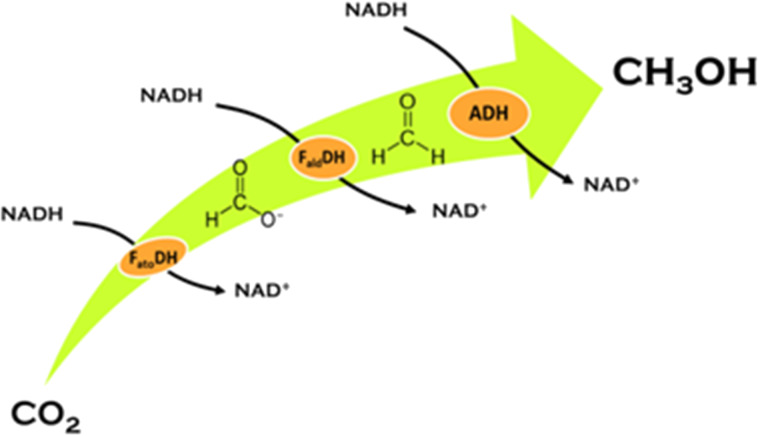
CO_2_ reduction to methanol in water promoted by F_ate_DH, F_ald_DH and ADH where three consecutive 2e^−^ steps are involved.

Currently, the enzymatic reduction of carbon dioxide is under investigation as a possible route for fuel production [4]. A specific application, which leads to the production of methanol, occurs in water through three 2e^–^ steps based on the use of three enzymes, namely: formate dehydrogenase (F_ate_DH), formaldehyde dehydrogenase (F_ald_DH), and alcohol dehydrogenase (ADH). These enzymes promote the cascade reduction of CO_2_ to methanol through formic acid (F_ate_DH), formaldehyde (F_ald_DH) and aldehyde (ADH). The reduction process is enabled by NADH, which is oxidized to NAD^+^. However, the production of one mole of CH_3_OH from CO_2_ requires the consumption of three moles of NADH (Figure 1).

Therefore, the regeneration of NADH is necessary for practical application of the described process. In nature, the endergonic process of NAD^+^ reduction to NADH is performed with the help of solar energy during photosynthesis. Presently, a substantial effort is being made for the regeneration of 1,4-NADH using a variety of strategies, including the use of enzymatic catalysis, as well as chemical, electrochemical and photochemical methods [5–6]. For industrial application, such reduction would require implementation of the most energetically and economically convenient technologies, such as visible-light-driven photocatalysis, as chemical methods are either too expensive or not compatible with the enzymes [7]. To date, photocatalysis has shown great potential for photodegradation of environmental pollutants [8–9]. Most interesting is that photocatalysis may play a relevant role in the conversion of large quantities of CO_2_ into fuel by using water or waste organics as the hydrogen source [7,10–11]. Therefore, integrated photochemical CO_2_ reduction/organic oxidation and H_2_O splitting have received significant interest due to their potential environmental and resource preservation benefits [12–13]. Interestingly, the oxidized forms of some common waste organics may find practical application.

The use of heterogeneous photocatalysts in the process of NADH regeneration from NAD^+^ would be of great interest due to their low cost, moderate (ambient) operational conditions and acceptable environmental impact. The most extensively applied photochemical processes are based on the use of TiO_2_ as a photocatalyst in oxidation reactions [14–16]. While pure TiO_2_ has a band gap energy of 3.2 eV (which is not compatible for use with visible light), modified TiO_2_ is known to be more suitable for carrying out photocatalytic processes utilizing the visible part of the solar spectrum [17].

In previous work, systems based on stable, encapsulated enzymes [7] for the enzymatic reduction of CO_2_ to CH_3_OH in water combined with the near-UV–vis light driven photoregeneration of NADH for increasing the CH_3_OH/NADH molar ratio was described [7]. However, this approach is a hybrid process involving: the enzymatic reduction of CO_2_ to CH_3_OH promoted by the reduced form of cofactor NADH, and the in situ photocatalytic reduction of NAD^+^ to NADH under visible-light irradiation, using semiconductors in water/bioglycerol mixtures. Bioglycerol is being produced in increasing volumes for bio-diesel production from oleaginous seeds. New applications for this product are being investigated [7,11,18–19] and its use as a H-source providing its oxidized derivatives (or even C_2_ molecules [11]) may be an interesting path for the economically viable use of large volumes of CO_2_. Our ultimate goal is to attain a highly efficient and selective reduction of NAD^+^ to 1,4-NADH or other equally active isomers upon visible-light irradiation in order to make the enzymatic reduction of CO_2_ to CH_3_OH viable.

## Results and Discussion

**Figure 2:**
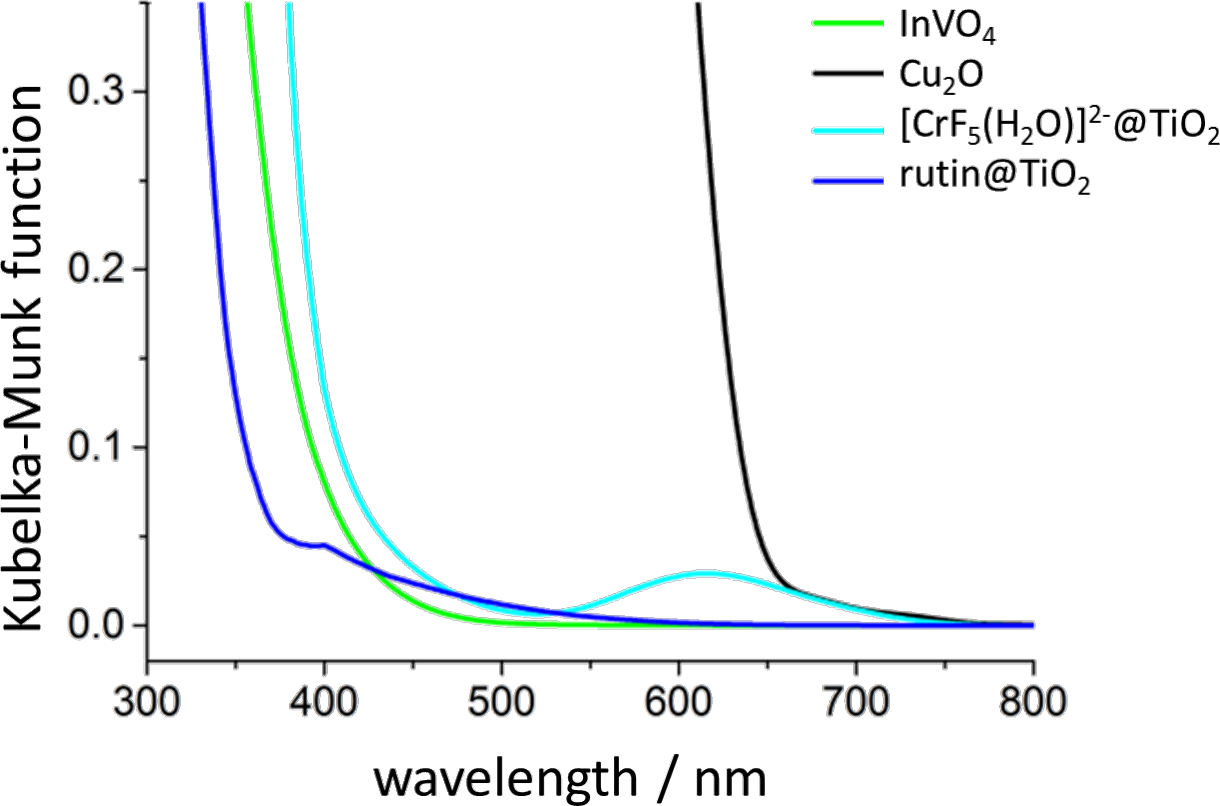
Transformed diffuse reflectance spectra of photocatalysts used in the present study.

In previous work [7] we demonstrated that encapsulated F_ate_DH, F_ald_DH, and ADH enzymes are able to rapidly (<1 min) reduce CO_2_ in proton–donor solvents under pH-controlled conditions, resulting in CH_3_OH at room temperature. The regeneration of NADH was attempted using both chemical and photochemical techniques. The former affects the enzymes that are quickly deactivated, while the latter techniques are more interesting and produce up to a few mol of methanol per mol of NADH (with respect to 3 NADH per methanol) as shown in Figure 1. As previously discussed [7], the success of the regeneration depends on the separation of the enzymatic reduction from the photoregeneration of NADH, thus a two-compartment reactor was used (see the Experimental section). In fact, the light most likely affects the enzyme activity by inducing structural modifications. In [7] a photocatalyst was used that operates on the border of the UV–vis spectrum. As described above, the goal of this research is to work in the visible-light range, possibly using direct irradiation with solar light. Therefore, we have synthesized a number of semiconducting materials showing photocatalytic activity under visible-light irradiation. The most active material was selected and fully characterized regarding its photoelectrical- and chemical-properties. Optimal conditions for use with visible light for the reduction of NAD^+^ using bioglycerol as a H-donor and a Rh(III)-complex as an e^−^–H^+^ transfer agent were found. It was shown that the photocatalyst, the electron mediator and the H-donor have suitable energy levels that can be combined together for an effective recycling of NAD^+^. The cofactor can be used several times in combination with the encapsulated enzymes, which promote the reduction of CO_2_ to methanol. In this work we discuss in detail the utilization of a new TiO_2_ photocatalyst (Degussa P25 or 10 nm particles produced in-house [20]) modified with the inorganic complex [CrF_5_(H_2_O)]^2−^ [20]. Additionally, the properties of other photocatalysts (Cu_2_O, InVO_4_, and TiO_2_, which are less active than the Cr-modified TiO_2_ photocatalysts), which were modified with the organic compound “rutin”, are briefly presented. Figure 2 shows the transformed diffuse reflectance spectra of the photocatalysts converted by the Kubelka–Munk function.

The properties of the photocatalysts used can be summarized as follows. Cu_2_O is a visible-light-absorbing, red-colored, *p*-type semiconductor with the band gap energy equal of 2.1 eV. InVO_4_ is an *n*-type semiconductor (*E*_bg_ = 2.8 eV). TiO_2_ modified with the organic compound rutin (rutin@TiO_2_) shows an electron injection into the conduction band of TiO_2_ as the result of a direct molecule-to-band charge transfer (MBCT) within the surface-formed, colored, charge-transfer complex of titanium(IV) [21]. TiO_2_ with an adsorbed chromium(III) anionic complex [CrF_5_(H_2_O)]^2–^ acts as a photosensitizer by injecting electrons into the conduction band of titania [20].

**Figure 3:**
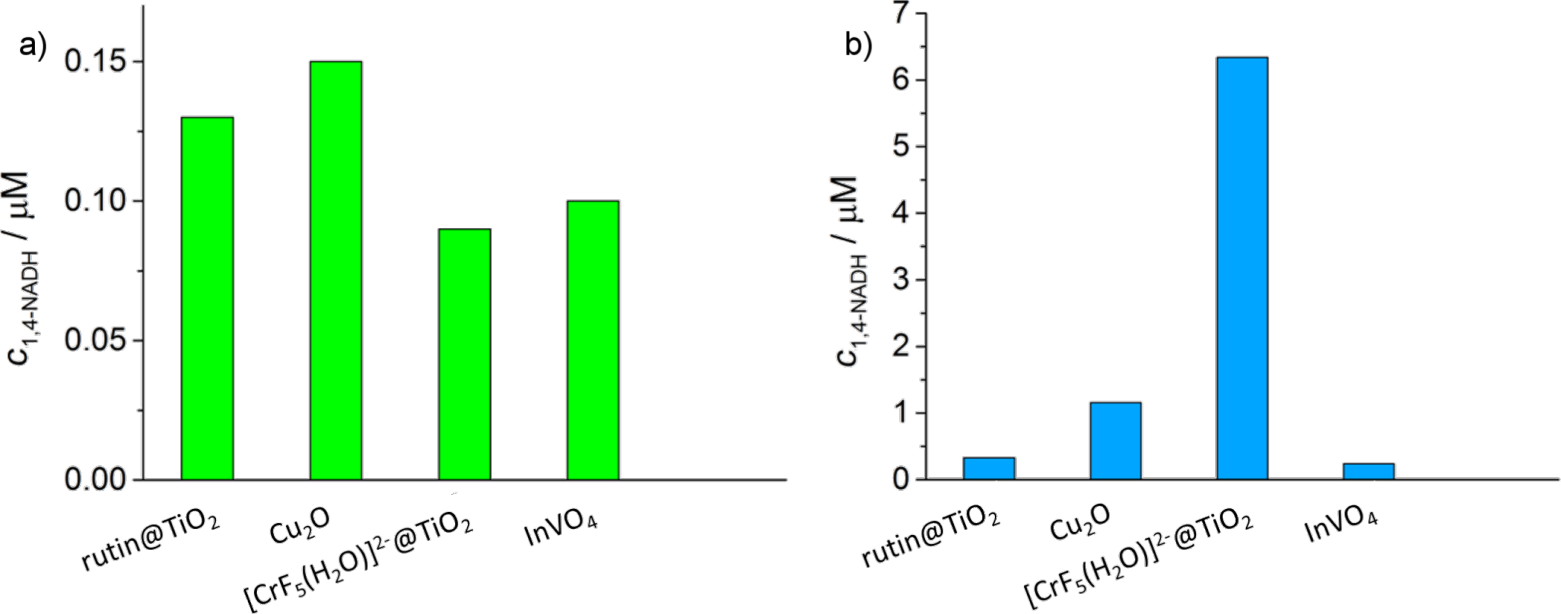
a) Photoregeneration of 1,4-NADH using water as an electron donor: after 6 hours of irradiation of the NAD^+^/water solutions in the presence of various photocatalysts. b) Photoregeneration of 1,4-NADH using several photocatalysts, and [Cp*Rh(bpy)H_2_O]Cl_2_ as an electron mediator or an H^−^-transfer agent. Note that the addition of glycerol to water improves the conversion yield and accelerate the reaction (see below).

Photocatalytic tests of NADH regeneration using these materials have been performed using visible light irradiation (λ > 400 nm). The results are summarized in Figure 3.

Figure 3a shows that the conversion yield of NAD^+^ into NADH is similar for the various photocatalysts. Additionally, the selectivity towards 1,4-NADH appears to be not very high. The concentration of the photogenerated, reduced form of the cofactor after 6 hours of visible-light irradiation in the presence of water is within the range of 0.1–0.15 μM. Noteworthy is that no or negligible reaction was observed when any of the components of the system was missing: photocatalyst, light, NAD^+^, or water. TiO_2_ alone was tested as a reference material yielding only minor traces of a complex mixture of products under the same experimental conditions. HPLC in combination with NMR analysis was employed for the detection of the various isomers of the NAD^+^-reduction products, namely the 1,4-NADH, 1,2-NADH, 1,6-NADH, or dimeric species. We have observed that when the photocatalysts alone were used, the selectivity towards 1,4-NADH was significantly lower than 100%. In fact the low amount of 1,4-NADH reported in Figure 3a is due to NAD^+^ conversion resulting in a mixture of compounds (including dimers) with a low selectivity (approximately 5%) towards 1,4-NADH. This is most likely due to the fact that the reactions take place on the surface of the photocatalyst without any selectivity. Conversely, the regeneration of 1,4-NADH was much more efficient via an indirect route of H^+^–e^−^ transfer, using hydride-transfer agents coupled to the photocatalytic materials. To implement such a strategy, the above described photoactive materials were coupled to the well-known [22] [Cp*Rh(bpy)(H_2_O)]Cl_2_ [aquo(2,2’-bipyridine)(pentamethylcyclopentadienyl)]rhodium(III), where Cp* = pentamethylcyclopentadienyl. For comparison we have also used its iridium analog, with phenantroline as a bidentate N-ligand replacing bpy. Iridium showed interesting activity, comparable to that of Rh. A key point in our approach was to demonstrate that the H^+^–e^−^ transfer system (photocatalyst, transition metal complex) we designed had an ideal potential for e^−^ transfer and could operate in combination with the H-donor and the enzyme to eventually convert CO_2_ into CH_3_OH. The success of this system was evidenced by measuring the amount of photogenerated 1,4-NADH using water or water/glycerol as electron donor. Figure 3b illustrates the results of the photocatalytic regeneration of NADH in presence of various materials after 6 hours of irradiation. Interestingly, for [CrF_5_(H_2_O)]^2−^@TiO_2_ the yield is 70 times higher in the presence of the electron mediator (under the same experimental conditions). The Cr-modified TiO_2_ showed the highest activity among the tested photocatalysts [20] and thus, the focus of the discussion is now shifted to this photocatalyst. Although CrF_3_-doped TiO_2_ is reported in the literature [8] to be active in oxidation processes of waste organics, neither TiO_2_ loaded with anionic [CrF_5_(H_2_O)]^2−^ nor CrF_3_ on TiO_2_ (used for reduction purposes) has been described so far.

**Figure 4:**
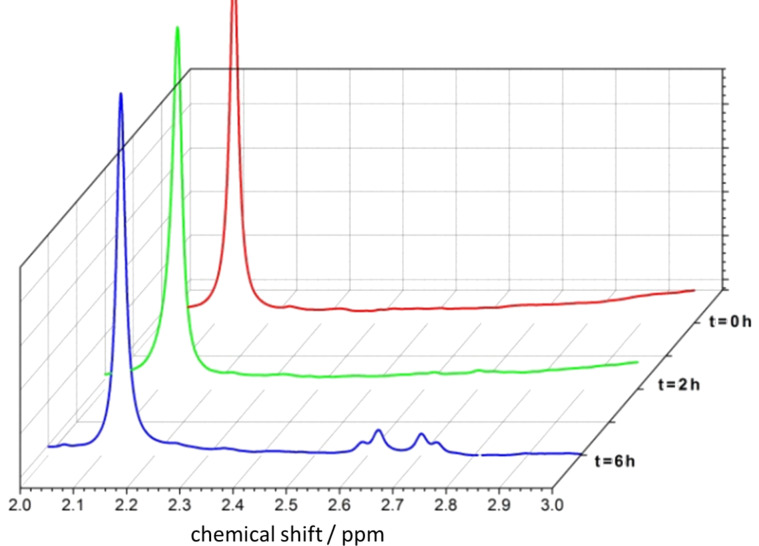
^1^H NMR spectra recorded at *t* = 0, after 2 and 6 h of irradiation in water. The selected range, 2–3 ppm, is diagnostic for NADH H-signals. The signal at 2.1 ppm results from the ribose hydrogen in the cofactor molecule and is taken as a reference peak. The concentration of the rhodium complex is 0.5 mM, with *c*_glycerol_ = 0.05 M.

The ^19^F NMR spectrum confirms the presence of the anionic form on the TiO_2_ surface with a signal at 121 ppm, while free [CrF_5_(H_2_O)]^2−^ shows a signal at 122 ppm (see Experimental section). In contrast, CrF_3_ has signals in a completely different region (−127 and −128 ppm). Therefore, we conclude that the supported form of Cr on TiO_2_ is the anionic complex [CrF_5_(H_2_O)]^2−^. Another issue in this process was to demonstrate whether the reduction is selective towards 1,4-NADH and no other isomers or if dimers were formed. The answer to this question was provided by using ^1^H and ^13^C NMR in connection with HPLC. The anionic pentafluorochromate-modified TiO_2_ coupled to the Rh(Ir)-transition metal complex appeared very selective towards 1,4-NADH, while neither isomers nor dimers were formed. Figure 4 shows typical ^1^H NMR spectra recorded during the course of the reduction: a pseudo-quartet centered at 2.65 ppm increases with time showing that the reaction occurs.

**Figure 5:**
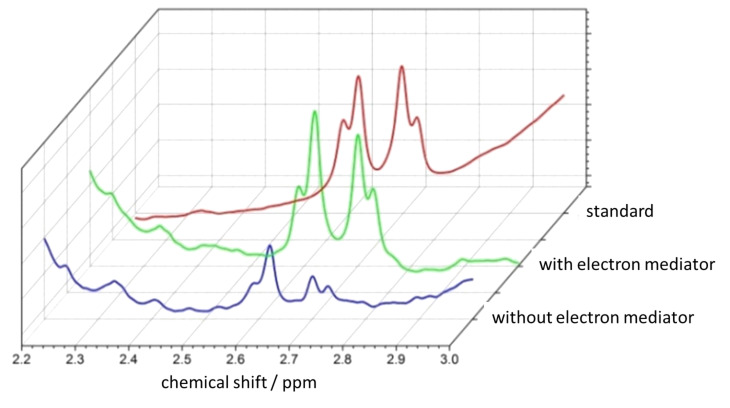
^1^H NMR spectrum of a standard 1,4-NADH (red line), and of 1,4-NADH formed from NAD^+^ upon photocatalysis in the absence (blue) and the presence (green) of the Rh-complex as a mediator and [CrF_5_(H_2_O)]^2−^@TiO_2_ as a photocatalyst.

This signal is due to 1,4-NADH, as shown in Figure 5. Here, the ^1^H NMR spectrum of standard NADH (a commercially available product) is compared with the spectra of the reduction products formed in presence and absence of the hydride-transfer agent used together with the [CrF_5_(H_2_O)]^2−^@TiO_2_ photocatalyst. The green and blue spectra were taken after 6 h of irradiation with solar light or white light under the same operative conditions with and without the Rh complex. They show that the presence of the Rh mediator improves the conversion rate.

It is known that the reduction of the [Cp*Rh(bpy)(H_2_O)]^2+^ **1** complex to [Cp*Rh(bpy)] **2** adds a proton and results in the conversion into a hydrido form. This product is an efficient and selective reduction catalysts of NAD^+^ to 1,4-NADH [22]. The resulting active hydrido form, [Cp*Rh(bpy)H]^+^ **3**, transfers a hydride ion to the 4-position of NAD^+^ (coordination to the amide-carbonyl-O-atom) thereby exclusively forming the enzymatically active, reduced 1,4-NADH. The purported mechanism, based on the rhodium complex, has been proposed elsewhere [16,23], and is shown in Equations 1–3.

[1]
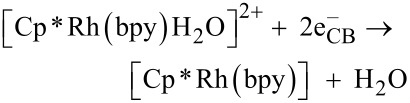


[2]
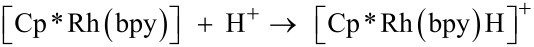


[3]
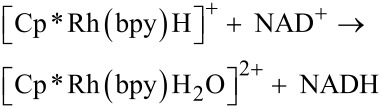


**Figure 6:**
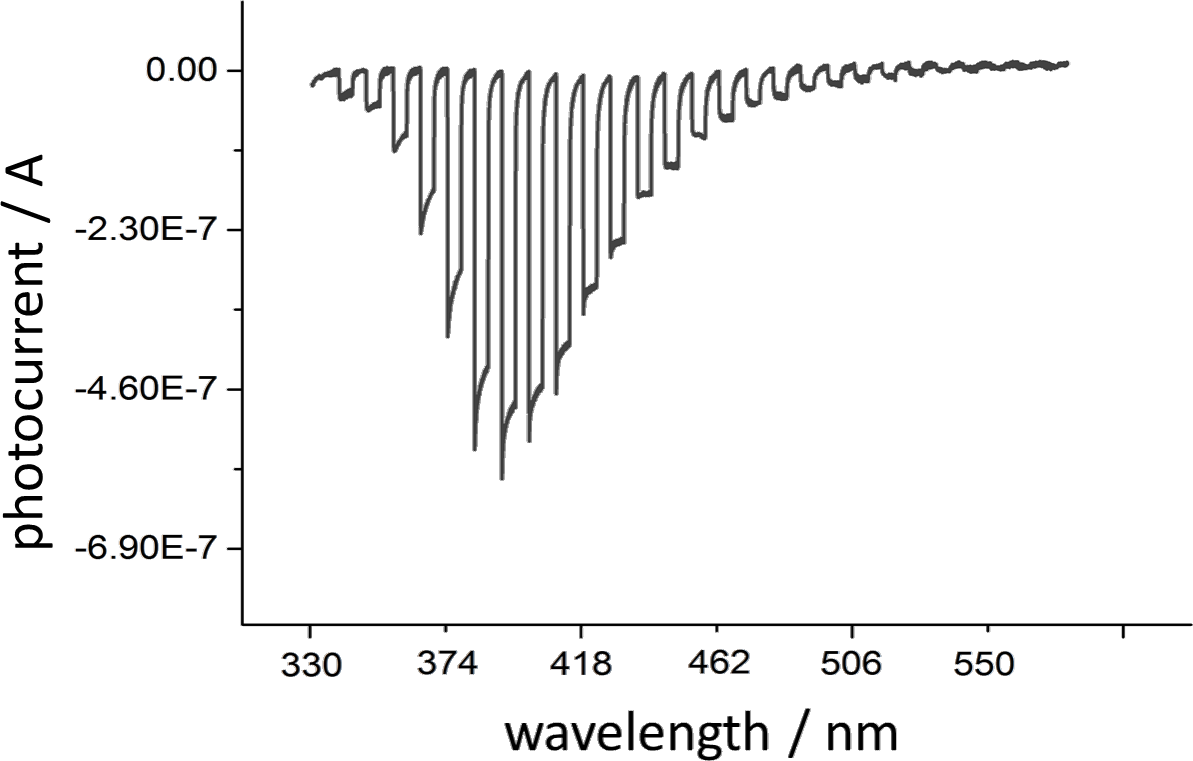
Photocurrent generated at the [CrF_5_(H_2_O)]^2−^@TiO_2_ electrode as a function of the wavelength of the incident light, recorded at constant potential of 500 mV vs Ag/AgCl. The spikes originate from the opening and closing of the shutter.

We have carried out dedicated experiments to confirm that such a mechanism holds in our conditions, and that the e^–^-transfer is thermodynamically and kinetically possible. This enables identification of the intermediates in the reaction pathway of the photocatalytic cycle based on [CrF_5_(H_2_O)]^2−^@TiO_2_ as an exciton generator and confirmation that the rhodium complex is an e^−^-transfer agent. The redox potential of the [Cp*Rh(bpy)H_2_O]^2+^/[Cp*Rh(bpy)H]^+^ couple was determined by Steckhan et al. and was shown to be equal to −0.32 V vs NHE. The redox potential of the conduction band of [CrF_5_(H_2_O)]^2−^@TiO_2_ is −0.58 V vs NHE, as measured in the present study using a previously published methodology [24]. The electrode covered by [CrF_5_(H_2_O)]^2−^@TiO_2_ generates a photocurrent upon visible light irradiation, proving a photoinduced electron transfer from the excited chromium(III) complex to the conduction band of TiO_2_ (Figure 6). The following step, that is, the transfer of electrons from the conduction band of the photocatalyst to the oxidized form of the rhodium complex (according to Equation 1), is thus thermodynamically feasible.

**Figure 7:**
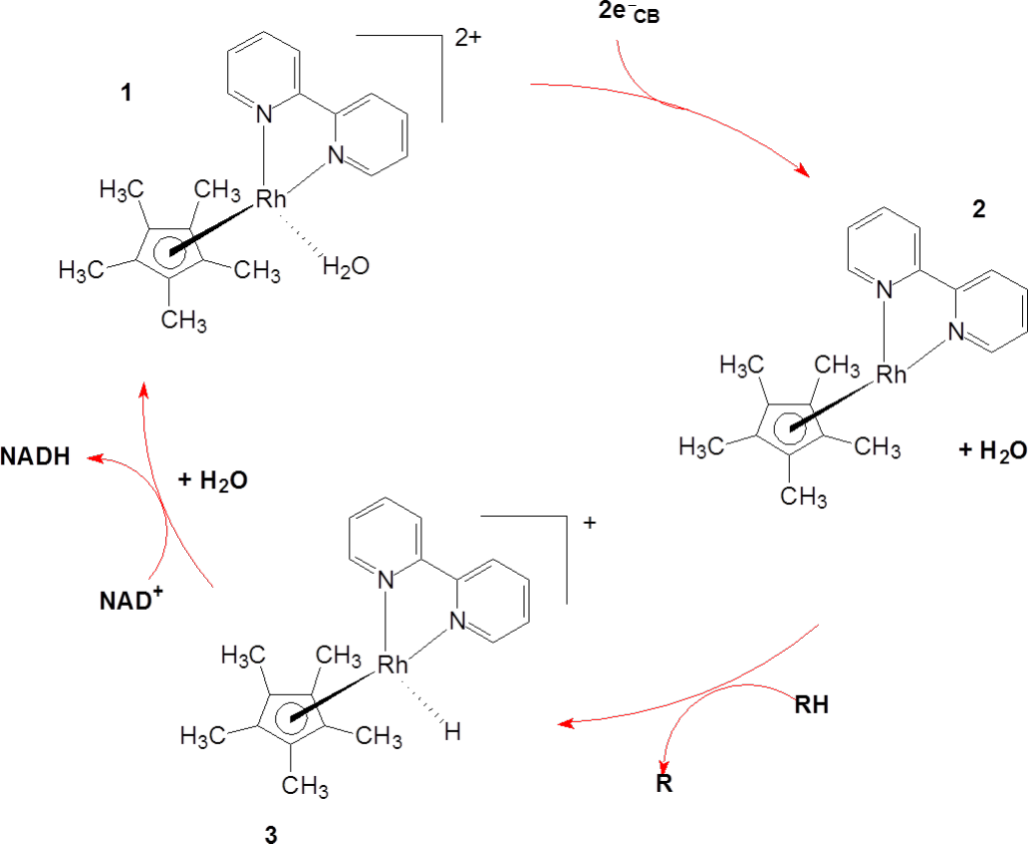
Expected role of the rhodium complex as an electron mediator.

The photogenerated holes can regain electrons via the oxidation of glycerol. The reduced complex (Rh(I)) reacts with a proton yielding a Rh(III)-hydrido species (Equation 2). The resulting Rh-hydrido-species transfers the hydride to NAD^+^ affording NADH (Equation 3 and Figure 7).

**Figure 8:**
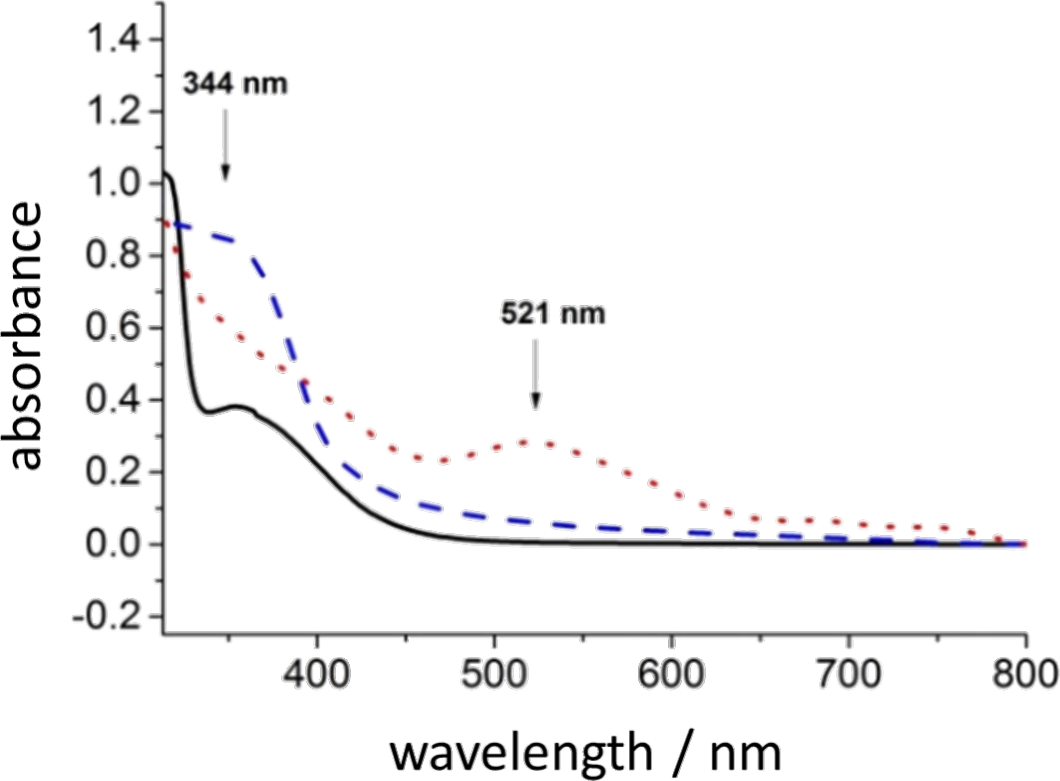
UV–vis absorption spectra of an aqueous solution of [Cp*Rh(bpy)(H_2_O)]^2+^. Continuous black line: spectrum of the starting rhodium complex in water. Dotted red line: spectrum of the rhodium complex after treatment with hydrogen. Dashed blue line: spectrum of the rhodium-hydrido complex after addition of NAD^+^: the band at 521 nm disappears and the band at 344 nm attributed to NADH appears.

Such steps, already hypothesized in the literature [23,25], are clearly demonstrated in the present work through the following experiments. First, [Cp*Rh(bpy)H_2_O]^2+^ was converted into [Cp*Rh(bpy)H]^+^ upon reaction with elemental hydrogen. The UV–vis absorption spectrum recorded after the reaction shows the appearance of a band at 521 nm that is characteristic of the formation of the rhodium hydride. This was confirmed by taking the spectrum of the isolated complex. The addition of NAD^+^ resulted in NADH formation (a band at around 344 nm) in concurrence with the disappearance of the 521 nm band (Figure 8). The formation–disappearance of the hydride was further confirmed by ^1^H NMR where a signal at −7.5 ppm (in the same region as the analog [Cp*Rh(6,6’-dimethyl-2,2’-bipy)H]^+^ [22]) was evident. This ^1^H NMR signal was correlated with the disappearance of the 521 nm band in the UV–vis spectrum, along with the appearance of the characteristic band at approximately 344 nm. The process was cyclic and the appearance–disappearance of the hydride signal followed the change in position of the UV–vis band from 521 to 344 nm and back.

**Figure 9:**
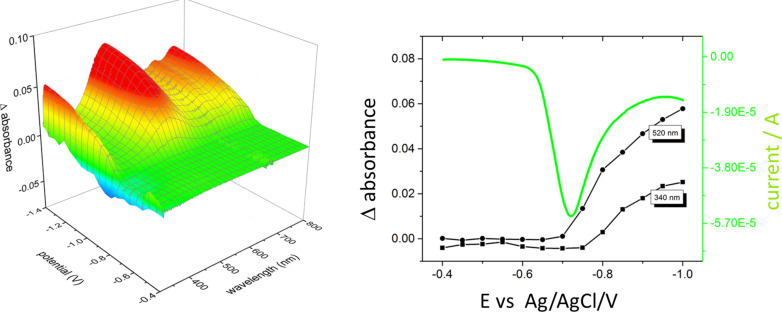
Spectral changes of the [Cp*Rh(bpy)(H_2_O)]^2+^ solution as a function of the applied potential (left). Cyclic voltammogram and corresponding changes in absorbance recorded at 521 nm (right). For simplicity, only the part of the potential scan (the reduction process from −0.4 to −1.4 V and back) is shown.

The spectral changes in the spectrum of [CrF_5_(H_2_O)]^2−^ are reported in Figure 9 together with the cyclic voltammograms.

The new combined system described in this paper has very high activity and specificity upon visible light irradiation. The extraordinary activity of [CrF_5_(H_2_O)]^2−^@TiO_2_ can be explained by an efficient photoinduced electron transfer from Cr(III) to the conduction band of TiO_2_ and further to the adsorbed rhodium complex. A hindered back electron transfer from the Rh species to the photocatalyst can also be responsible for the overall efficiency of the process. Substitution of Rh with Ir and of bipyridine with phenanthroline did not improve the yield to appreciable extent, thus the Rh-complex was used. Other photomaterials which are active upon visible light irradiation were also tested, such as Fe/ZnS, Co/ZnS, Ag/ZnS, ZnBiO_4_, AgVO_4_, NiO, CrF_3_@TiO_2_, tiron@TiO_2_. However, they did not show significant activity in the NADH photoregeneration process when compared to the [CrF_5_(H_2_O)]^2−^@TiO_2_ photocatalyst.

**Figure 10:**
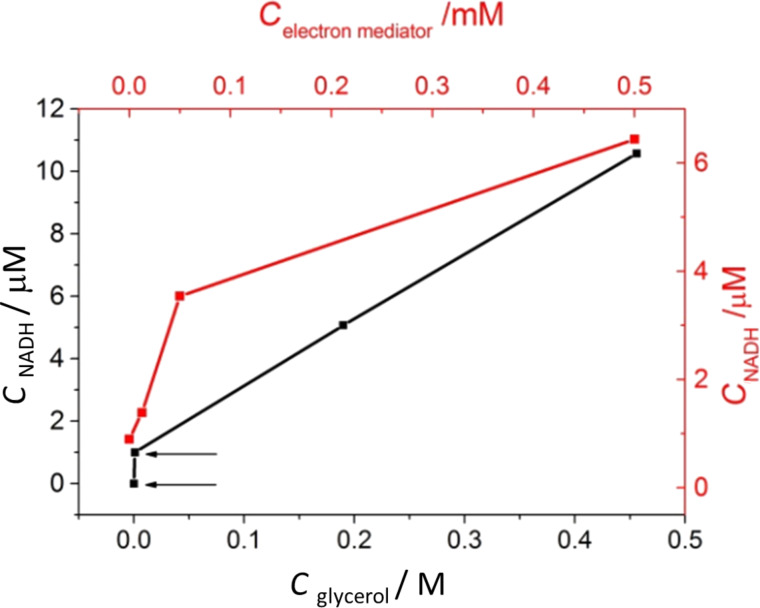
Photoreduction of NAD^+^ as a function of concentration of glycerol (black line) and [Cp*Rh(bpy)H_2_O]Cl_2_ (red line) under visible light irradiation in the presence of [CrF_5_(H_2_O)]^2−^@TiO_2_ (irradiation time; 6 h).

As mentioned above, glycerol was considered as an electron donor in aqueous solution. When only water was used in the photoreduction, the reaction rate was very low due to the weak ability of H_2_O to transfer electrons. Figure 10 shows the influence of the nature of the electron donor and the concentration of the electron mediator, on the reduction rate.

The NADH regeneration rate exhibits a strong dependence on the concentration of glycerol, which plays the crucial role of a sacrificial electron donor. Figure 10 also shows that the increase of the glycerol concentration from 0 M (the lowest point on the black line in Figure 10) to 0.001 M (the second point in the same figure) results in an extremely rapid increase of the reaction rate. It must be emphasized that the reaction in the presence of other electron donors, such as triethanolamine or isopropanol, was also tested but were not comparable with glycerol. For practical applications the mixture of glycerol 0.1–0.5 M in water was used.

Figure 10 shows also the influence of the concentration of [Cp*Rh(bpy)H_2_O]Cl_2_ on the reduction of NAD^+^ to 1,4-NADH. The rate greatly increases with an increasing concentration of the electron mediator.

**Figure 11:**
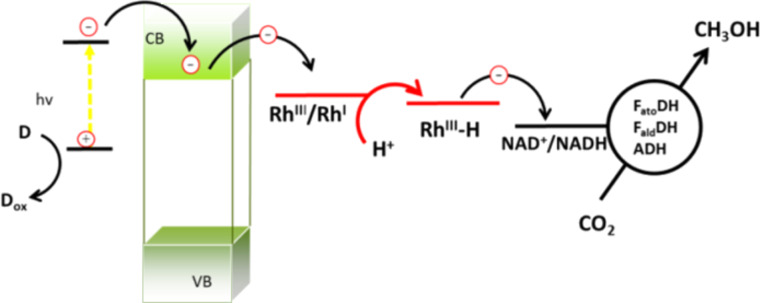
The electron flow in the photocatalytic system of NAD^+^ reduction composed of the photosensitized TiO_2_ photocatalyst, Rh(III)-electron mediator and glycerol as a sacrificial reagent.

These findings demonstrate that the photocatalysts described in this paper are able to utilize visible light for the generation of the electron–hole couples (exciton). The excited electrons are transferred to reaction centers at the surface of the photo-materials, and the conversion of NAD^+^ to NADH occurs, involving the electron mediator and the oxidation of glycerol. The overall mechanism is summarized in Figure 11.

The first and only product of glycerol oxidation is 1,3-dihydroxyacetone (1,3-DHA), as demonstrated by NMR studies carried out on a fresh reaction mixture (^1^H resonances found at 4.34 ppm, as compared with literature data: ^1^H at 4.40 ppm) [26]. The dihydroxyacetone species is not very stable and can be easily converted into other monomeric or polymeric species under the reaction conditions. Oxidation of glycerol is still under investigation as the isolation of 1,3-DHA would add value to the process. Other parameters, such as the optimization of the photosystem for a more efficient NAD^+^ reduction, the improvement of the reaction selectivity, the increase of the absorption coefficient, and the increase of the chemical stability and photostability of photocatalysts, are also under continuous investigation for their further improvement and bring the whole reaction closer to a potential application.

## Conclusion

Titanium dioxide modified with chromium(III) complex, [CrF_5_(H_2_O)]^2−^@TiO_2_, exhibits great ability to drive the in situ selective reduction of NAD^+^ cofactor to 1,4-NADH. This process works particularly well in the presence of a [Cp*Rh(bpy)H_2_O]Cl_2_ complex playing the role of the electron transfer mediator. Our studies demonstrate that the photocatalyst is able to utilize visible light for generation of the electron–hole couples. The excited electrons are transferred to an e^−^-transfer mediator at the surface of the materials, where the conversion of NAD^+^ to NADH occurs, involving the eventual oxidation of glycerol. The overall mechanism is summarized in Figure 11.

Anodic photocurrents generated by the photocatalyst upon visible light irradiation suggest the electron transfer from the excited sensitizer (chromium(III) complex) to the conduction band of TiO_2_. The reduction of the rhodium complex was confirmed by spectroscopic studies made in the presence of irradiated TiO_2_ and upon electrochemical reduction of the complex. Selective reduction of NAD^+^ to 1,4-NADH has also been experimentally evidenced by ^1^H NMR and HPLC measurements. Finally, the enzymatic route of the CO_2_ reduction was confirmed to occur as was previously described [7,11]. The novelty of the photochemical NADH regeneration described in this paper consists of the use of visible light and the coupling of an inexpensive photocatalyst with robust e^−^- and H^−^-transfer mediators, which can be used for days. Furthermore, we have demonstrated the entire mechanism of the electron flow from the photo-excited photosensitizer to NAD^+^, resulting in NADH generation, which can be further used in enzymatic processes including carbon dioxide reduction.

The product of glycerol oxidation is 1,3-dihydroxyacetone. This species is not very stable and can be converted into other monomeric or polymeric species. Oxidation of glycerol is still under investigation together with other parameters, such as the optimization of the photosystem for a more efficient NAD^+^ reduction, the improvement of the reaction selectivity, the increase of the absorption coefficient, and the increase of the chemical stability and photostability of photocatalysts.

The photocatalytic system discussed in this paper is much more effective as compared to ZnS-based photocatalysts as previously presented in [7,11] and represents a significant step towards potential application of this hybrid technology for CO_2_ reduction to methanol.

## Experimental

### Preparation of photocatalysts

#### Synthesis of photosensitized TiO_2_

The modification of TiO_2_ with rutin was carried out as reported in the literature [27]. A titanium dioxide powder, P25 Evonik (500 mg), was added to 10 cm^3^ of aqueous rutin solution (10^−2^ mol dm^−3^). The suspension was sonicated (10 minutes) and the colored precipitate was collected, washed 3 times with water and dried in air at 60 °C.

[CrF_5_(H_2_O)]^2−^@TiO_2_ was prepared by impregnation of TiO_2_ particles (P25, Evonik or 10 nm particles) [20] by (NH_4_)_2_[CrF_5_(H_2_O)] under ultrasonic stirring. The suspension was sonicated for 15 minutes, left for 24 hours, and the nanoparticles were isolated washed with water and dried under vacuum.

Copper(I) oxide was prepared in the reaction of an aqueous solution of glucose (10 mL, 0.8 M) dropped into an alkaline solution of CuSO_4_ (50 mL, 0.2 M) in presence of polyvinylpyrrolidone K-30 (0.3 g) at 80 °C. After 1 hour a red precipitate was separated by filtration, washed with water and dried.

Vanadates were prepared as reported by Hu et al. [28] with minor modifications: NaOH and V_2_O_5_ powders in a molar ratio of 6:1 were dissolved together in water and stirred. Subsequently, the solution of In(VO_3_)_3_ or AgNO_3_ was added. Precipitates, which appeared immediately, were aged at room temperature for 1 hour, washed and dried.

#### Synthesis of (NH_4_)_2_[CrF_5_(H_2_O)]

5 mL of an ammonia solution was added to 50 mL of an aqueous solution of NH_4_·HF (15 g). A CrF_3_ solution was added dropwise to a hot (363 K) solution of NH_4_·HF in the presence of zinc powder. A green precipitate was obtained, isolated and analyzed, resulting in the title compound.

#### Synthesis of [Cp*Rh(bpy)H_2_O]Cl_2_

[Cp*Rh(bpy)H_2_O]Cl_2_ was obtained from [Cp*RhCl_2_]_2_ as reported in [29]. Phenanthroline was used instead of bpy to generate [Cp*Rh(phen)(H_2_O)]Cl_2_. The analogous Ir complex was prepared starting from [Cp*IrCl_2_]_2_. The hydride [Cp*Rh(bpy)H]^+^ showed a hydride signal at −7.5 ppm in its ^1^H NMR spectrum.

#### UV–vis characterization

The UV–vis diffuse reflectance spectra of the photocatalysts were recorded using a UV-3600 spectrophotometer (Shimadzu) equipped with an integrating sphere. Powder samples were ground with BaSO_4_ (1:50 wt ratio). Barium sulfate was used as a reference.

#### Regeneration of NADH from NAD^+^

Photocatalytic tests of NADH regeneration were performed in a borosilicate glass reactor (*V* = 10 mL). The photocatalyst (1 g L^−1^) was suspended in deoxygenated phosphate buffer (pH 7). NAD^+^ (0.8 mM) was added. Glycerol was used as an electron donor. [Cp*Rh(bpy)H_2_O]Cl_2_ was used as electron mediator (concentration range: 0–0.5 μM). The suspension was irradiated in the sealed reactor, under nitrogen atmosphere, using a 50 W LED illuminator (λ > 400 nm) as light source or concentrated solar light. 2 mL samples were collected during irradiation, filtered and analyzed by HPLC (column Zorbax SB-Aq) and NMR.

#### Reduction of CO_2_ to CH_3_OH using the assembled photocatalytic/enzymatic system

**Figure 12:**
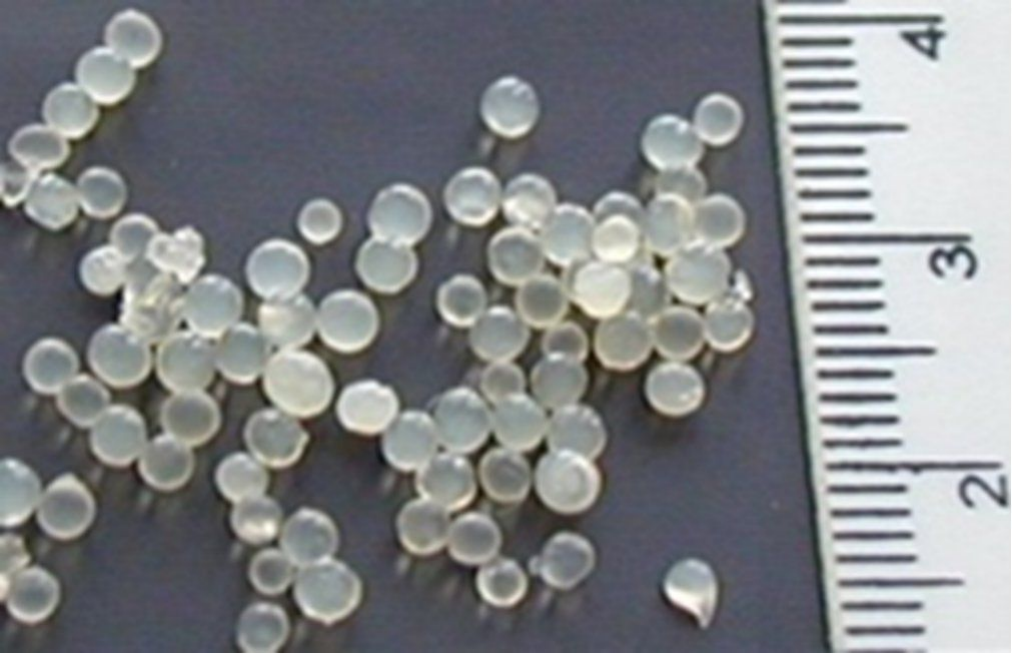
Beads produced from Ca-alginate and TEOS containing co-encapsulated F_ate_DH, F_ald_DH and ADH.

**Figure 13:**
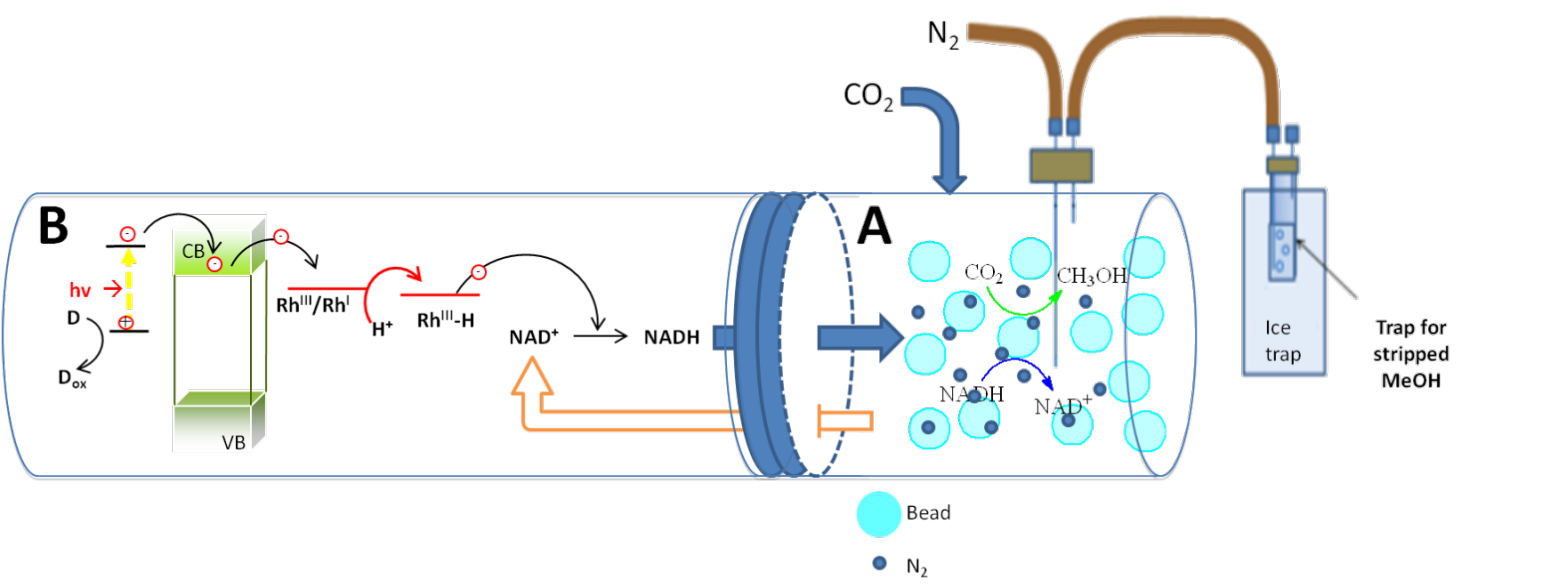
Assembled photocatalytic/enzymatic system for reduction of CO_2_ to CH_3_OH.

The reduction of CO_2_ to methanol using encapsulated enzymes is described in [7]. Here we recall that the enzymes F_ate_DH, F_ald_DH, and ADH were encapsulated into silicate cages made from Ca alginate and tetraethoxysilanes (TEOS) and used at a controlled pH of 7. CO_2_ was admitted in a continuous flow reactor at such a rate to generate a 20% excess with respect to the stoichiometric amount required by the enzymes. Excess CO_2_ was avoided to prevent its emission into the atmosphere since a goal of the research is to reduce CO_2_ emissions during the synthesis of methanol. For this reason, the recovery of methanol by stripping was carried out using N_2_ and not CO_2_ itself, which would also be possible. A more complex experiment with CO_2_ recovery is currently under investigation, so that CO_2_ can be used as reagent and carrier of methanol. In this study, air influenced the enzymes and was not used. The beads (Figure 12) were suspended in water in compartment A of the reaction cell below (Figure 13) in presence of the required amount of NADH, and CO_2_ was slowly admitted.

When the formation of methanol (monitored by GC on withdrawn samples of the reaction solution, within 1 min maximum) was at the maximum, the solution was pumped into compartment B, which contained the photocatalyst and the heterogenized Rh complex, while the encapsulated enzymes remained in compartment A. Irradiation (0.5–1 h) with visible light (or solar light) caused the conversion of NAD^+^ into NADH to occur at the maximum yield (very close to 100%). The solution was again pumped into compartment A where the reduction of CO_2_ occurred with formation of CH_3_OH. The cycle was repeated until no more CH_3_OH was formed. At given intervals, CH_3_OH was extracted in compartment B (stripping was realized by bubbling N_2_ and condensing of the vapors) to avoid an increasing concentration which might block the enzymes. The difference in the rate of the enzymatic reaction and the photocatalytic regeneration of NADH is a barrier to practical utilization of this hybrid technology. Further efforts for improving the convergence of the time of reaction in the two steps is currently underway.
